# Stimuli Responsive Ionogels for Sensing Applications—An Overview

**DOI:** 10.3390/membranes2010016

**Published:** 2012-02-07

**Authors:** Andrew Kavanagh, Robert Byrne, Dermot Diamond, Kevin J. Fraser

**Affiliations:** CLARITY—The Centre for Sensor Web Technologies, National Centre for Sensor Research, School of Chemical Sciences, Dublin City University, Dublin 9, Ireland; Email: andrew.kavanagh@dcu.ie (A.K.); Robert.Byrne@dcu.ie (R.B.); dermot.diamond@dcu.ie (D.D.)

**Keywords:** stimuli responsive polymers, ionogels, ionic liquids, hybrid materials, molecular photoswitches, solid state electrolytes

## Abstract

This overview aims to summarize the existing potential of “*Ionogels*” as a platform to develop stimuli responsive materials. Ionogels are a class of materials that contain an Ionic Liquid (IL) confined within a polymer matrix. Recently defined as “a solid interconnected network spreading throughout a liquid phase”, the ionogel therefore combines the properties of both its solid and liquid components. ILs are low melting salts that exist as liquids composed entirely of cations and anions at or around 100 °C. Important physical properties of these liquids such as viscosity, density, melting point and conductivity can be altered to suit a purpose by choice of the cation/anion. Here we provide an overview to highlight the literature thus far, detailing the encapsulation of IL and responsive materials within these polymeric structures. Exciting applications in the areas of optical and electrochemical sensing, solid state electrolytes and actuating materials shall be discussed.

## 1. Introduction

The concept of a chemical sensor is one in which a material is used as a sensing agent and exhibits a selective interaction with a target species or analyte [1,2]. The specific interaction between the sensor and analyte produces a signal, which can then be observed via an appropriate detection scheme [3,4]. Liquid based sensors can suffer from volatility and handling issues, meaning their performance suffers over time. Producing solid-state platforms is of great importance for some applications, as the solid-state removes many of the issues associated with that of the liquid state [5]. There is great interest therefore in solid-state chemical sensors that can provide reliable signals at a low unit cost, and through careful optimization of the sensitive polymer composition, prevent leaching or removal of key components over time [6]. Polymer gels have been employed in sensing templates for this purpose and we will explore their use in detail in this review [7,8].

A polymer gel is defined as an interconnected polymer network formed within a liquid phase [9,10]. When the polymer network is generated in the presence of an Ionic Liquid (IL), the resultant gel has been termed an *ionogel* within the literature [11]. Ionogels are therefore a new class of hybrid material that combine the physical properties of both the polymer gel and the physically entrapped IL within [12]. 

A recent review of the area focused on the differing subclasses of polymers employed (*i.e.*, organic, inorganic and hybrid repeating units), and the modes of preparation of ionogels [13]. Previous reviews focused on the interaction and mobility of the IL within the polymer network [14]. This overview will complement these reviews by focusing on the application of ionogels as functional materials for direct application as sensing and actuation agents. Publications detailing the response of ionogels to changes in pH, metal ion chelation, incident electromagnetic radiation and interactions with biomolecules will be discussed. Our group in particular have detailed the use of ionogels to produce opto/electronic sensors for transition metal ions [15,16], as the basis of electrochromic devices [17], as actuating materials for controlling fluid movement [18] as reference electrodes [19], and building biosensing platforms [20] This overview will present the current status of the use of ionogels in these areas, beginning first with an introduction to the area of ILs themselves. 

## 2. Ionic Liquids

According to current convention, a salt melting below the normal boiling point of water is known as an IL, thus forming liquids that are comprised entirely of cations and anions at room temperature [21]. In contrast to conventional organic liquids/solvents, important physical properties of ILs such as viscosity, density, melting point and conductivity can be tuned to suit a particular need by the appropriate choice of the cation/anion combination [22]. ILs typically contain a large bulky asymmetric cation together with a smaller π-delocalized anion which overwhelmingly exhibit electrostatic interactions; thereby preventing the formation of a structured lattice [23,24]. ILs exhibit good thermal stability [25], are intrinsically conductive [26] and have been shown to have electrochemical windows as high as 5.0 V in some cases [23]. 

These unique properties are of benefit for chemical sensing applications, as a good knowledge on the overall chemistry of the IL can be used to improve on the limitations of previous sensing approaches.

### Synthesis of ILs

**Figure 1:**
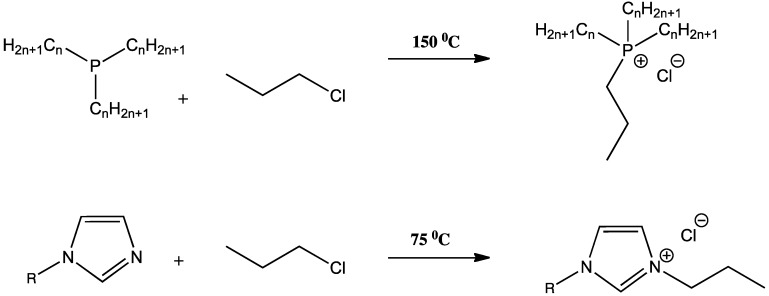
Direct nucleophillic addition of a trialkylphosphine **(top) **and 1-substituted imidazole **(bottom) **with 1-chloropropane to form their corresponding chloride Ionic Liquids (ILs).

The current literature is dominated by two main cation-families, ammonium and phosphonium based ILs. The quaternization of phosphines has been described in the literature by Bradaric *et al.* [27] and Ermolaev *et al.* [28], whilst the corresponding reaction for amines is described by Busi *et al.* [29]. The reaction schemes are somewhat similar in that both electron rich starting materials undergo a S_n_2 addition reaction in the presence of a haloalkane at elevated temperatures over time. A general reaction scheme for the quaternization of a trialkylphosphine with 1-chloropropane to form the phosphonium salt is presented in Figure 1 (top); whilst the quaternization of 1-substituted imidazole with 1-chloropropane to form the imidazolium salt is shown in Figure 1 (bottom). Both reactions can be undertaken without solvent, as the reactants are liquid in both cases. Once the reaction is complete, the new IL formed is easily isolated by vacuum removal of the volatile halo-alkanes.

Variants of the halide ILs can be prepared via their ion-exchange metathesis reaction with group I organic anions [30,31]. Key to the success of this reaction is the choice of solvent that it is undertaken in, it must serve to solvate the new IL formed and *preferably* promote the precipitation of the by-product (a classical alkali-halide salt). As the IL begins to form over time, so too do the ionic interactions of alkali and halide atoms, respectively, which transfer out of the reaction solvent. The new IL can then be isolated by filtration of the by-products and drying over time.

**Figure 2:**
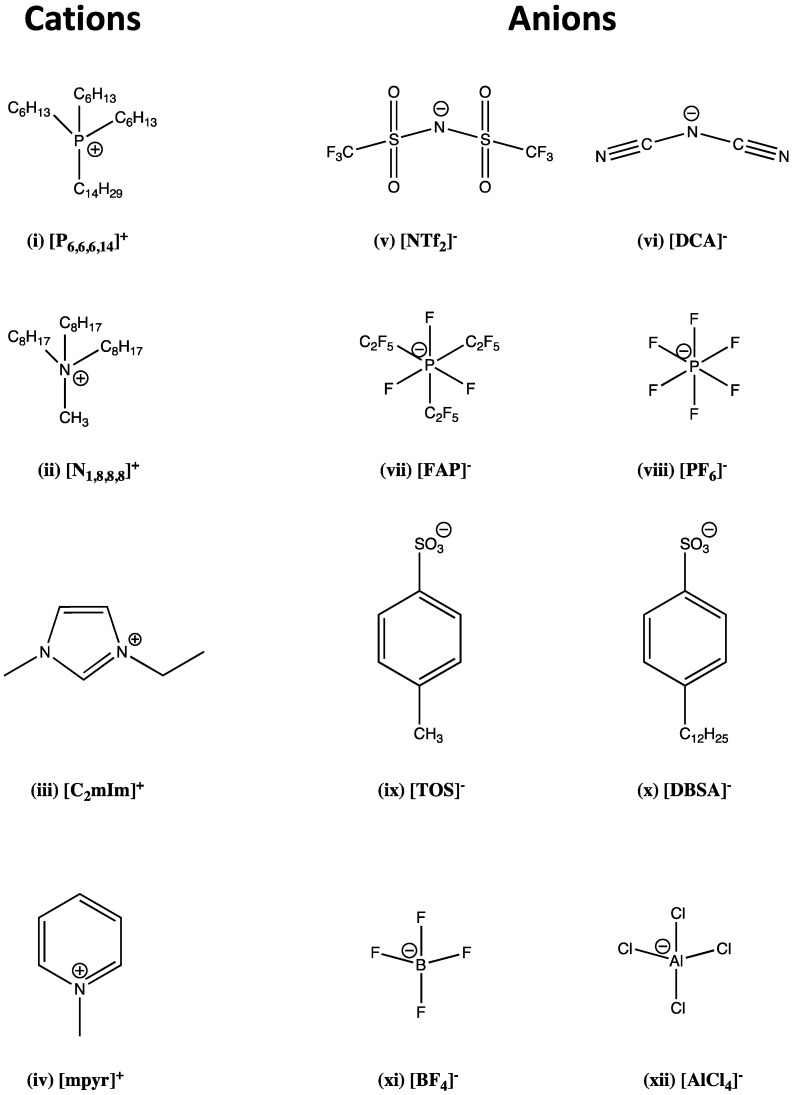
A list of the more popular ions used throughout the literature for IL syntheses. Cations are listed on the left [(i)–(iv)], and anions are listed on the right [(v)–(xii)].

A list of the of the more popular ions produced via quaternization and ion-exchange reactions can be seen in Figure 2; beginning with the tetra-alkylated phosphoniums and ammoniums [(i) and (ii)], *N*-heterocyclic amines [(iii) and (iv)], the amides [(v) and (vi)], hexafluorinated phosphates [(vii) and (viii)], benzenesulfonates [(ix) and (x)] and finally some group III tetrahalides [(xi) and (xii)]. 

## 3. Stimuli Responsive Materials

Stimuli responsive materials (SRM’s) are those that can undergo (in some cases a reversible) change in their molecular configuration in response to an externally applied stimulus. They can of course be subdivided in relation to the particular stimulus applied, and are the subject of previous reviews. These reviews have discussed the alteration of molecular configurations in response to irradiated light [32], to an applied voltage [33], to a change in temperature [34], to an incident magnetic field [35] or a change in the pH of the surrounding environment [36]. The change in molecular configuration is usually accompanied by an observable signal, for example a change in color, conductivity or surface energy. They are therefore ideal candidates for use as chemical sensors; if the molecular rearrangement can be induced by an interaction with a defined analyte, then it can also be used as the chemical sensor signal. Photo responsive materials are particularly good candidates for chemical sensors as, in some cases, the incident irradiation can be low enough in power (such as in the use of low power LEDs [37,38]) to be non-invasive on the sensing materials. This is an important consideration for materials that may be subject to photostability issues, as exposure to high power sources can lead to rapid decomposition of the material. Addressing this issue can lead to improved reproducibility in the response obtained, which will improve the performance of the sensing device over increased time periods [39]. 

### Photo-Responsive Materials

**Figure 3:**
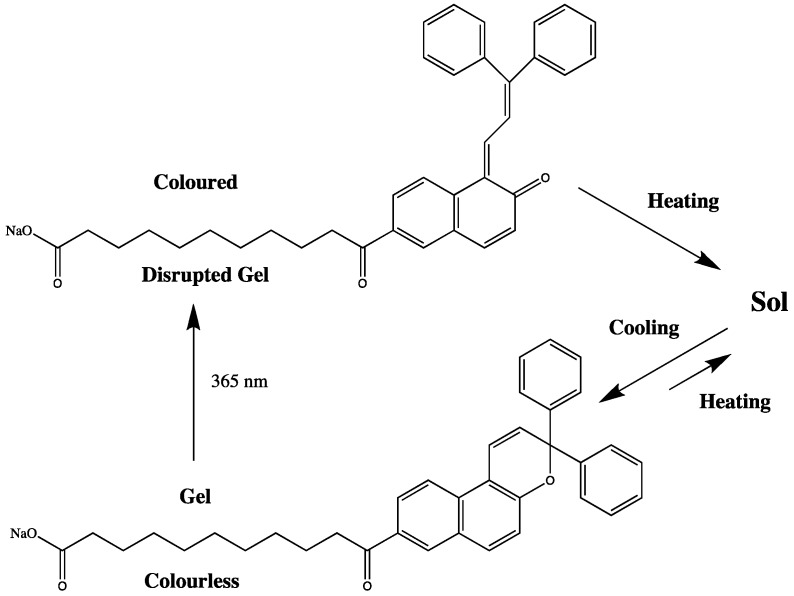
Light-induced switching between open and closed isomers of naphthopyrans [48].

The *cis-trans* isomerization of azo compounds and stilbene has also been used to produce photo-responsive solutes for switching the viscosity of the resulting solution [40,41]. Azobenzenes are particularly good candidates for use as chemical sensors as the photoisomoerization event not only yields a change in color [42], but also a significant change in polarity [43,44]. The use of organogelators (a molecule which exhibits significant electrostatic interactions leading to the formation of an interconnected network [45,46]) has proved a worthy route for the development and incorporation of the photo-responsive chemistries of azo compounds into the solid state. Murata *et al.* [47] developed a cholesterol-based gelator containing azobenzene; irradiation of the system at 330–380 nm led to isomerization of the *trans* azo linkage to its *cis* conformation and thereby disrupting gelation. Irradiation of the sol at wavelengths longer than 460 nm resulted in reversal of the isomerization and re-formation of the gel. Pozzo and co-workers [48] employed the photochromic equilibrium of 3,3-diphenyl-*3*H-naphthopyran, which brings about a large conformational change. Two forms of the naphthopyran can be distinguished: a colored open form and a colorless closed form [48]. In the closed form the naphthopyran unit does not influence the stacking process and the molecule acts an efficient gelator. Irradiation with UV light converts the naphthopyran unit into the open form and prevents the carbamate group from stacking. The isomerization is accompanied by a color change, and while the gel liquefies it becomes yellow. Heating converts the pyran unit back into the closed form, and upon cooling a colorless gel is again obtained (Figure 3).

**Figure 4:**
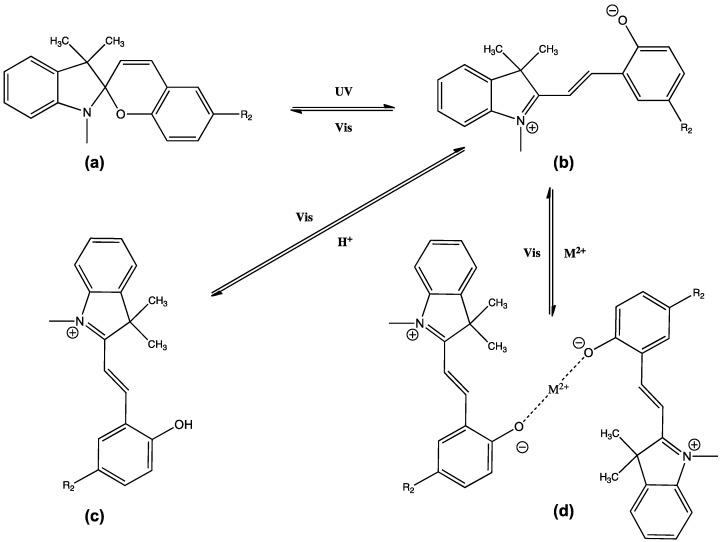
The photodynamic, pH and metal-ion chemistry of Spiropyran (SP) materials; **(a)** SP, **(b)** Merocyanine (MC), **(c)** MC-H and **(d)** MC-M^2+^ complex.

Of particular interest to many are the photo-responsive molecule Spiropyran (SP) and its zwitterionic isomer Merocyanine (MC) [49,50]. The two isomers exist in a photo-dynamic equilibrium controlled by the application of UV and visible light, respectively [51,52]. Furthermore, MC can act as a Lewis base in the presence of an acid or metal ion solution, forming protonated forms and ion-complexes. Particularly interesting is that the ion/proton binding can be reversed using white light, releasing the bound species and reforming the inactive SP isomer. In addition to the SP and MC isomers, the protonated and metal-ion chelated forms exhibit unique optical properties and the entire system is therefore inherently self-indicating of their status (Figure 4). 

Previous research has explored the behavior of the SP and MC isomers in systems capable of user controlled transition metal ion uptake and release [39,53], as effective solvatochromic probes [54,55] and as hybrid materials that exhibit user controlled multi-switchable optical properties [56]. Combining the photo-chemistry of SP and MC with the responsive chemistries of a hydrogel was first investigated by Szilagyi *et al.* [57]. We have expanded the area slightly to combine the properties of photo-responsive hydrogels with ILs producing ionogels [18,58], which will now form the basis of the next discussion. 

## 4. Stimuli Responsive Ionogels

### 4.1. Photo Responsive Ionogels for Direct Fluid Control in Microfluidic Devices

One of the issues for microfluidic or “lab on a chip” devices is the controlled movement of liquid throughout the device. The practical solution has been to employ high power consuming pumps and/or actuating valves, which means that most prototype devices are anything but a “lab on a chip”. One novel alternative to this approach, is to control fluid movement using stimulus-responsive polymer valves integrated into the fluidic system that can be controlled using light [18]. The valve was based on an ionogel of which there were two distinct components: **(a) **The polymer gel, a co-polymer based on (poly(*N*-isopropylacrylamide) (pNIPAAM) and SP, and **(b) **phosphonium ILs.

**Figure 5:**
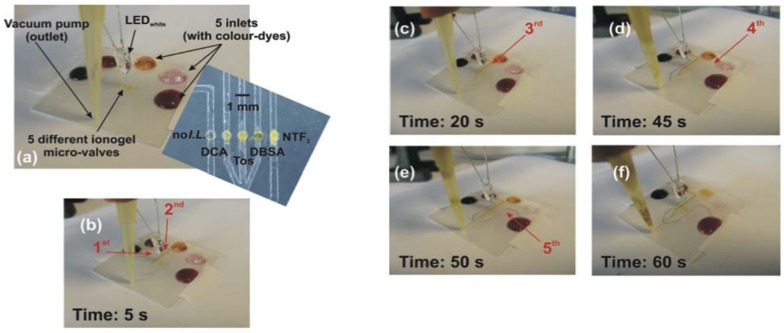
Overview of the photo-responsive ionogels: **(a) **micro-valves closed; the applied vacuum is unable to pull the dyes through the micro-channels; **(b)** white light is applied for the first time; **(c)–(f) **controlled fluidic flow as a function of the IL anion in the ionogel [18] (Reproduced by permission of The Royal Society of Chemistry).

pNIPAAM is a well known hydrogel that can swell and contract thereby drawing in and expulsing aqueous liquids as a function of pH and temperature [59,60]. In this work, the authors initially soaked the ionogels in acidic solution, which caused them to swell, and protonated the SP as part of the polymer backbone (Figure 4 (b),(c)). Figure 5 is a brief summary of the work, in which the ionogels were incorporated into a microfluidic device based on poly(methylmethacrylate) (PMMA). Four ionogel devices were prepared based on phosphonium cations with amide and benzensulfonate anions.

**Figure 6:**
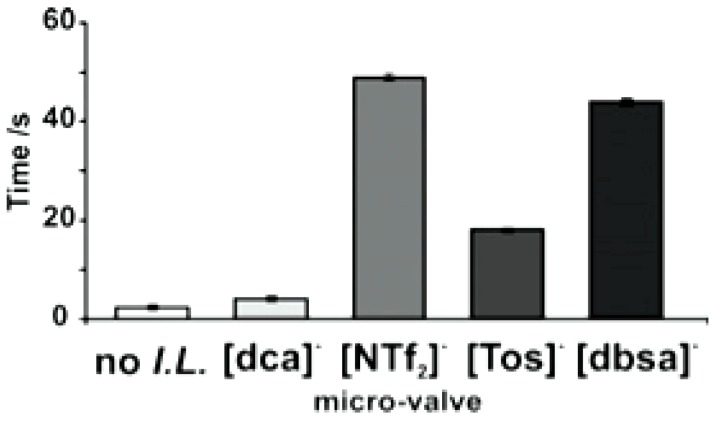
Actuation times of photo-responsive ionogel valves under uniform white light illumination as a function of the IL anion [18] (Reproduced by permission of The Royal Society of Chemistry).

Irradiation of the ionogels with white light caused the protonated MC isomer to revert back to its SP closed form. This induced a change in the polarity of the ionogel (hydrophilic, MC-H^+^ to hydrophobic, SP), and the subsequent expulsion of water from the gel, causing it to contract. The role of the IL proved crucial in controlling the rate of contraction (and hence the fluid movement) as the thermal relaxation of the MC-H^+^ to SP is significantly affected by its physico-chemical interactions with the IL [54,55]. Through variation of the ionogel composition; the micro-valves were tuned to open at different rates when illuminated under the same white light source (Figure 2 (c)–(f), and Figure 6.) 

### 4.2. Electro Responsive Ionogels for Sensing Applications

A particular advantage of using ionogels occurs in applications where the intrinsic ionic conductivity of the ionogel can be exploited, for example in electrochemical sensors and devices. Ion-selective electrodes (ISEs) are a particular breed of electrochemical sensor, which convert the activity of a given analyte in solution into a voltage potential. ISEs employ polymer gels based mainly on poly(vinylchloride) (PVC) and PMMA [61,62], for the selective detection of important environmental and biological analytes at levels as low as nanomolar concentrations in some cases [63,64]. As PVC and PMMA exhibit high glass transitions, organic plasticizers are used to produce flexible transparent polymer membranes. They are prepared by co-dissolution with a suitable molecular solvent, producing the membrane as the solvent evaporates [65]. The sensing agents are often incorporated into the polymer membranes via their co-dissolution with the polymer and the plasticizer solution.

A prerequisite for a plasticizer therefore is that it must exhibit a markedly lower glass transition itself. Phosphonium ILs have been shown to exhibit glass transitions as low as −77 °C [66], and have been shown to plasticize both PMMA and PVC, producing Тflexible films suitable for use in ISEs [67,68].

A particular advantage of using ILs as plasticizers for PVC/PMMA gels is that they can further replace some of components needed for a functioning ISE. For example, an ion-exchanging salt is typically used in ISE membranes to facilitate the movement of the analyte between the aqueous and polymeric phases [69]. Chernyshov *et al.* have reported the selective detection of dopamine and adrenaline at concentrations as low as 1 × 10^−7^ M, using an IL as plasticizer without the need for an ion-exchanging salt [70].

Ionogels have also recently found use in amperometric sensors. Nádherná *et al.* described the use of an ionogel based on poly(ethyleneglycol) and 1-butyl-3-methylimidazolium hexafluorophosphate [C_4_mim][PF_6_] as an amperometric sensor for nitrogen dioxide. Using the ionogel as the electrolyte, the authors reported a detection limit as low as 0.3 ppm [71]. 

### 4.3. Electro Responsive Ionogels for Electrochromic Applications

Electrochromic materials undergo a reversible change in their optical properties in response to an applied voltage [72]. They are good candidates for sensing templates as the color can vary by the choice of the electrochrome (of which there are many [73,74]) and its concentration. The color generated can therefore act as a sensor signal in response to an electrical potential [75]. Ionogels are good candidates for electrochromic devices as their intrinsic ionic conductivity can facilitate the charge required to produce the optical response [17].

A publication by Ahmad *et al.* describes the development of a complementary electrochromic device based on Tungsten oxide and Prussian blue [76]. The authors prepared on ionogel using sol-gel hydrolysis chemistry of Tetraethyl orthosilicate (TEOS) in the presence of the IL 1-ethyl-3-methylimidazolium bis(trifluoromethane)sulfonamide [C_2_mIm][NTf_2_], which formed a solid-state device upon solidification between two electrodes. In this case the responsive chemistry of the ionogel facilitated the current flow toward the electrodes (to which the chromophores were deposited), which in turn caused a change in the optical properties of the device. The authors reported an ionic conductivity of 1.2 × 10^−2^ S/cm for the ionogel, which allowed for fast switching times whilst the coloration efficiency of the device was calculated to be 64 cm^2^/C.

Recent publications have detailed the use of ionogels as the solid-state electrolyte for electrochromic devices based on 4,4’-disubstituted bypyridinium salts (viologens) [17,77], poly(aniline) [78], poly(pyrrole) [79] and Prussian Blue [80].

### 4.4. Optically Responsive Ionogels for Sensing Applications

**Figure 7:**
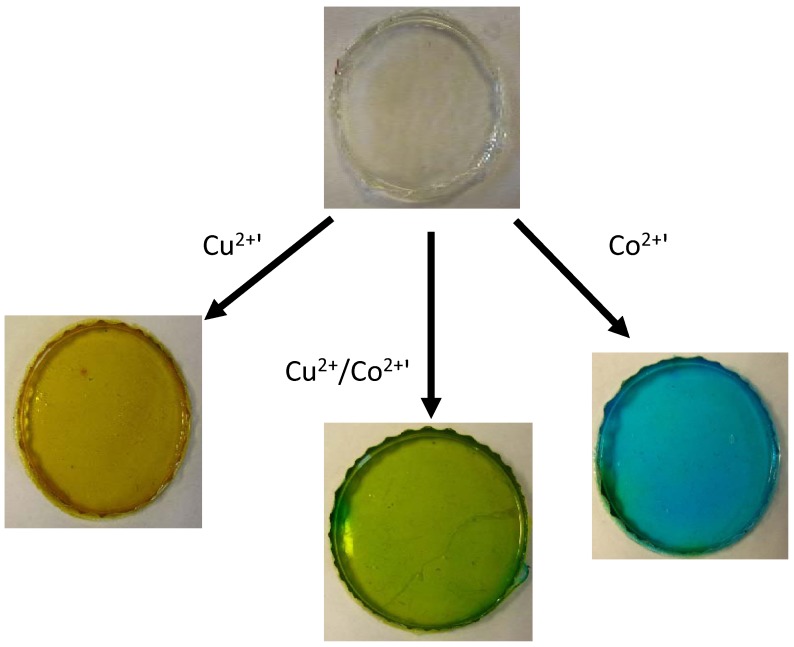
Optical response of *trihexyltetradecylphosphonium dicyanamide* ([P_6,6,6,14_][DCA)/poly(vinylchloride) (PVC) based ionogel upon complexation with Cu^2+^ (yellow, left), Co^2+^ (blue, right), and a mixture of the two (green, middle) [15].

Polymer optodes are similar to ISEs in terms of their composition, and how the analyte transfer is facilitated from the sample into the membrane phase [81]. Ionogels have been employed within optodes, mainly as the plasticizer to produce the film. Zhu *et al.* used phosphonium based ionogels with a conventional chromoionophore for the detection of important inorganic acids such as HCl and H_2_SO_4_ [82]. The authors detailed enhanced selectivity toward hydrophilic anions for the ionogels versus conventional plasticizers, which they have attributed to the increased dielectric constant of the ionogel. A publication based on a similar concept by Topal *et al.* detailed the increased selectivity of zinc phthalocyanines to pH when incorporated into imidazolium ionogels [83]. One particular publication on ionogels as optodes detailed significant template simplification (as a result of the particular IL used), whilst the ionogel/optode produced three distinct colors in the presence of Cu^2+^ and Co^2+^ ions [15] as shown in Figure 7.

Lunstroot *et al.* have pioneered the concept of ionogels as solid-state luminescence devices in two separate publications [84,85]. In both cases the ionogels were based on a siloxane support, whilst the IL was used to bind to lanthanide element that exhibited photoluminescence upon UV irradiation. In fact Cheminet *et al.* recently expanded the concept of optically responsive ionogels to fluorescent materials [86]. A phenylene–ethynylene oligomer was synthesized and chemically tethered to an imidazolium cation; which exhibited fluorescence in the solid state as part of a siloxane based ionogel. 

### 4.5. Ionogels as Bio-Sensing Components

Along with the electrochemical applications of ILs (as discussed), ILs have gained momentum in bio applications. Recent work in the area include ILs as biocatalytic reactions [87,88], biosensors [89], protein stabilization [90] and biopreservation [91]. They have been proposed as unique solvents for biomolecules such as proteins/enzymes because of their unusual solvation characteristics. Work by Fujita *et al.* [89,92] and others [90,93] have shown that some proteins are, in fact, soluble, stable and remain active in some ILs. As a case study Cytochrome c (cyt. c) was found to have enhanced solubility and stability in a biocompatible IL solution based on the dihydrogen phosphate anion [92]. This is an important observation since proteins are sometimes unstable when handled in vitro, and stabilizing agents are a necessary component to ensure their long-term stability. 

This is especially true of proteins that have pharmaceutical potential since lack of stability is a limitation to widespread use of some protein therapeutics. It has been well documented that enzyme performance in an IL is affected by several parameters including water activity, pH and impurities [94]. Other important factors that play a role in enzyme stability/activity include IL polarity, hydrogen bond basicity and nucleophillicity of anions, ion kosmotropocity and viscosity. 

Although outside the scope of this discussion, these areas have been discussed in an excellent review by Zhao [95]. Abe *et al.* [96] recently synthesized a number of phosphonium salts that have an alkyl ether group present. The phosphonium salts moiety is commonly found in living creatures, and it was hypothesized that this family of ILs have good affinity with enzyme proteins and may provide a good environment for enzymes. Some examples are based on the immobilization of enzyme-IL systems in chitosan [97] or Nafions [98]. Thus, catalytically active proteins and enzymes may also be confined for biosensors applications in order to achieve direct electron transfer within ionogels. 

It is therefore proposed that incorporating these biocompatible ILs into ionogels is a particularly attractive strategy in the field of biosensing. These materials, in theory, will inherit all of the favorable IL properties whilst being in a solid, gel like structure [99]. 

#### 4.5.1. The Need for Wearable Sensors

Wearable sensors allow the continuous monitoring of a person’s physiology in a natural setting. At present, health-monitoring systems using electronic textiles are mainly targeting applications based upon physiological parameter measurements, such as body movements or electrocardiography (ECG). However, due to their relative complexity, there is very little activity in the development of real-time wearable chemo/bio sensing for sports applications.

Micro-Total-Analysis-Systems (µTAS) and Lab-on-a-Chip (LOAC) technology are widely used in analytical chemistry and biotechnology [100] but they are still rarely used in other areas like sports science. In this field, wearable sensors are becoming increasingly employed, through the use of embedded transducers or smart fabrics for monitoring parameters like breathing rate, heart rate and footfall [101]. These sensors require that the desired sample of analysis, usually a body fluid such as sweat is delivered to the sensor’s active surface, whereupon a reaction happens and a signal is generated. Ideally, the system must be low cost, while still being robust, miniature, flexible, washable, reusable or disposable [1]. All these requirements point to micro-fluidic devices as the key tools for improving wearable chemo-/bio-sensing [102].

There are several factors that correlate sweat pH and health. Changes in the pH of the skin are reported to play a role in the pathogenesis of skin diseases like irritant contact dermatitis and acne, among others [103]. Patterson *et al.* [104] showed that inducing metabolic alkalosis through the ingestion of sodium bicarbonate led to increased blood and sweat pH. Furthermore, it has been reported that sweat pH will rise in response to an increased sweat rate [105]. A relationship was also observed between pH and sodium (Na^+^) levels in isolated sweat glands in that the greater the concentration of Na^+^, the higher the sweat pH will be [106]. As exercising in a dehydrated condition has been shown to lead to increased levels of Na^+^, it can be seen that such changes can be measured directly (e.g., using a Na^+^ selective sensor) or indirectly by monitoring sweat pH [107].

**Figure 8:**
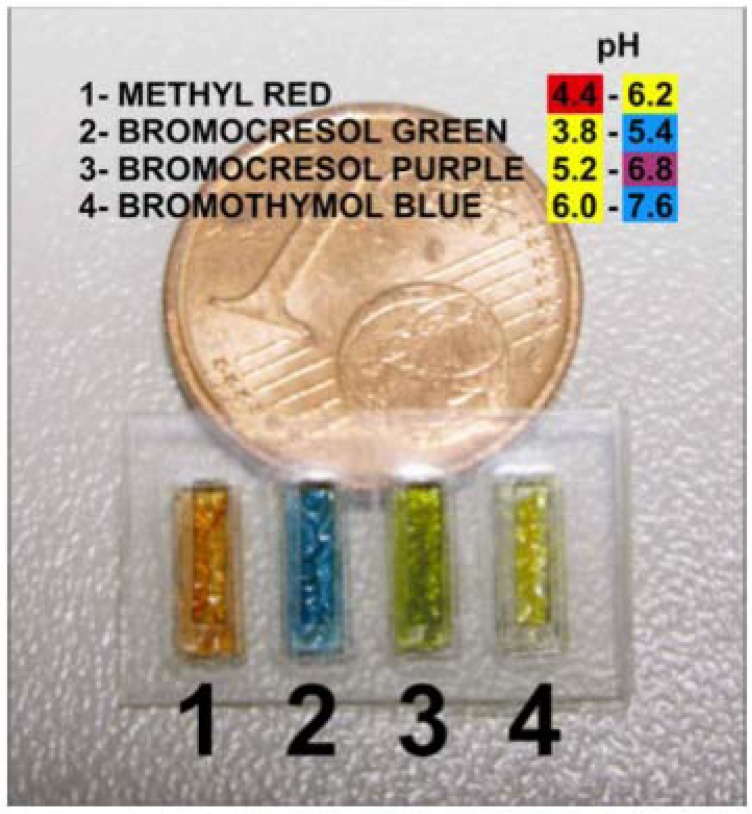
Barcode system for the analysis of human sweat developed by Benito-Lopez *et al.* [110]. Ionogels were based on pNIPAAM and Phosphonium ILs, whilst the change in optical properties of the encapsulated colorimetric dye was used to correlate with the pH of the incident sample.

In order to provide sensors with good sensitivity, selectivity and stability, various support materials, methods and reagents for immobilization of pH indicators were employed by our group [108,109]. A barcode system (as shown in Figure 8) was developed as an initial sweat sensor with an ionogel being an important component in the fabrication of the sensing platform [110].

The ionogel consisted of two monomeric units; (NIPAAm) and *N*,*N*-methylene-bis(acrylamide) (MBAAm) in the ratio 100:5, respectively [57]. The reaction mixture was prepared by dissolving both monomeric sub-units and the photo-initiator dimethoxy-phenylacetophenone (DMPA) into the IL([P_6,6,6,14_][DCA]). The barcode (18 × 10 mm), consisted of four independent reservoirs, and was easily fabricated in PMMA and pressure-sensitive adhesive in five layers using CO_2_ ablation laser [110]. Immersing the barcode in de-ionised water and then varying the pH from 0 to 14 in intervals of one pH unit, allowed the stability of the barcode to be studied. The pH was monitored using a benchtop pH meter and it was repeated at least four times without observing any damage to the barcode and/or ionogel. 

The ionogel matrix proved to be very robust even at harsh pH conditions (0–14) and it was shown that the pH indicators bromocresol green, bromocresol purple and bromothymol blue retained their pH indicator properties within the ionogel. The ionogel-dye interactions ensure no leaching of the dyes occurs during experiments, thereby providing long durability of the device for the monitoring of sweat pH measurements over time [110,111].

Important contributions to current sensor research in the area of LOAC or µTAS systems can be made by employing ionogels as active constituents within organic electrochemical transistors (OECTs). Many of the applications for OECT/ionogel sensors will likely involve disposable devices, cheap fabrication and therefore enzyme stability is of the utmost importance. OECT devices are inherently low-power and relatively easy to fabricate. 

Nilsson *et al.* [112] demonstrated the potential for OECT sensors to be manufactured inexpensively using printing techniques for mechanically flexible single-use tags. They fabricated humidity sensors on thin polyester foils and on paper. Combining OECT properties with those of ionogels therefore offers significant potential for realizing new generations of solid-state biosensing devices in a variety of form factors, and using ILs to optimize the stability and reactive nature of the host enzyme.

**Figure 9:**
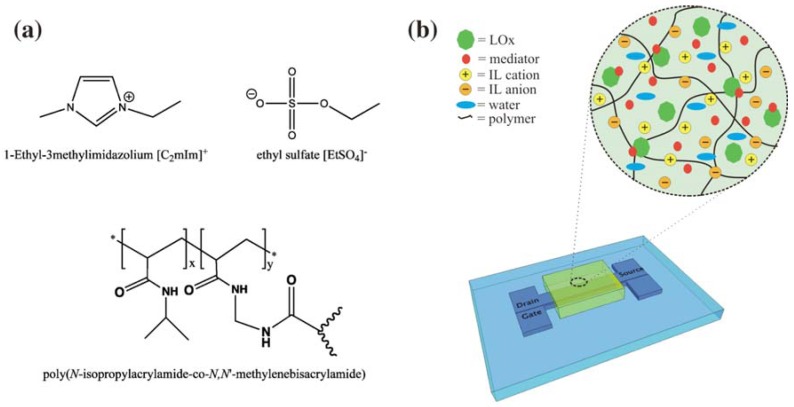
(**a**) Ionogel components and (**b**) a schematic representation of the organic electrochemical transistor (OECT) device with ionogel/enzyme mixture [20] (Reproduced by permission of The Royal Society of Chemistry).

Most recently the development of a solid-state electrolyte incorporated into an OECT has been reported for the detection of lactate [20]. The authors report the possibility of a fast, reliable, robust, and cheap way of measuring lactate concentration in physiological fluids using an ionogel as the basis of a biosensor (Figure 9) which will open the way to lactate biosensors for health and sport applications. Current methods of detection of lactate include conducting polyaniline films [113], carbon nanotubes [114], screen printed Prussian blue electrodes [115], and biosensors based on electro-chemiluminescent detection [116]. Commercial lactate sensors are also available [117], based on standard electrochemical methods. One example is the lactate SCOUT (Senslab), which however, performs measurements directly in blood, making real-time detection invasive and impractical for long-term studies. 

The detection of lactate (deprotonated form of lactic acid) in blood provides a biochemical indicator of anaerobic metabolism in patients with circulatory failure [118]. In addition to its presence in blood, lactate can be found in sweat (concentration range between 9 to 23 mM), reflecting, in an indirect way, eccrine gland metabolism [119]. It is well known that lactate concentration increases during physical exercise, making it a useful parameter to monitor wellness, physical fitness and the effects of exercise [120]. 

**Figure 10:**
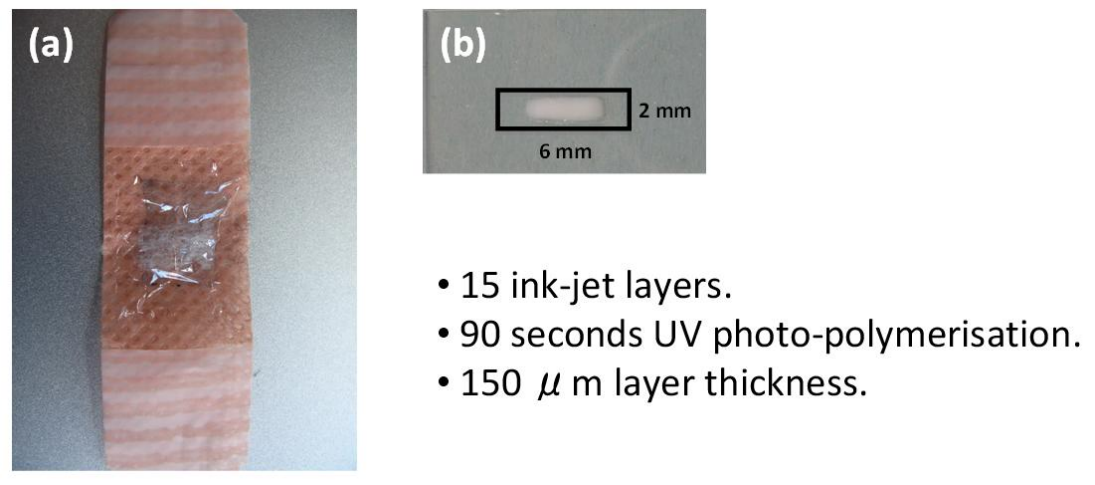
A printable OECT/Ionogel composition incorporated into a widely available fabric (plaster) and the development of printable ionogels. Volumes are in the nL range.

The possibility to pattern PEDOT:PSS in a wide variety of substrates such as glass, flexible plastic and textiles, opens new routes for the development of wearable biosensors. Such biosensors can therefore be incorporated into fabrics such as t-shirts, sweat bands or shorts allowing for the analysis of real time measurements of the target biomolecules. Figure 10, (a) shows a proof of concept of this type prepared in our laboratories which consists of an OECT transistor incorporated into a plaster, while (b) shows an ionogel formulation printed using an inkjet printer (volume in the nano liter range). However, more studies are needed to understand device degradation mechanisms and improve sensor lifetimes. 

## 5. What Does the Future of Stimuli Responsive ILs Hold?

### 5.1. PH, Photo-Responsive ILs

As the explosion of interest in ILs as diverse, functional agents for multidisciplinary research continues, so too has the advent of ILs exhibiting stimuli responsive chemistries. This represents a subsection topic for both respective fields of research (ILs and SRM’s). Previously solid, lattice structured and anionic SRMs have been shown to form liquids when their respective counter ions are exchanged for those used in IL studies (Figure 2 (i)–(iv)). The new materials therefore exhibit their previous responsive chemistries in the liquid phase, combined with properties inherent to ILs (negligible vapor pressure, ionic conductivity, *etc.*). Recently, two individual communications described ILs based on the photo and pH responsive chemistries of azobenzene. 

Pina *et al.* first described the synthesis of ILs based on the azobenzene dye Methyl Orange (MO) [121]. ILs were prepared by allowing the sodium salt of MO to undergo ion-exchange metathesis with halide ILs of imidazolium, phosphonium, sulfonium and guanidinium respectively, yielding intrinsically photochromic ionic liquids. The paper details how the behaviour of photochromic ILs can be tuned by choice of the cation, as measured by the individual thermal relaxation rates from the *cis* back to *trans* structure of four different ILs dissolved in water and ethanol. 

**Figure 11:**
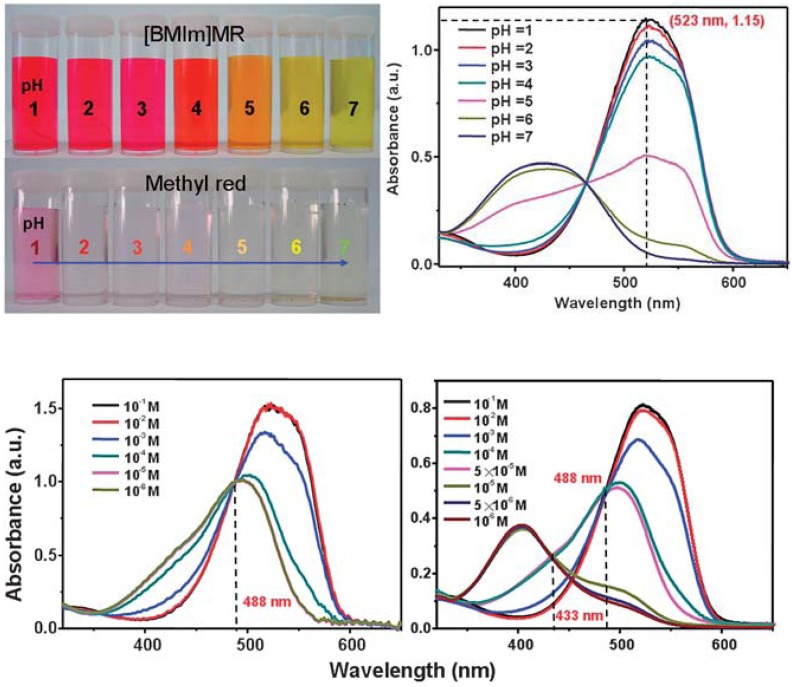
**(Top)** Increased selectivity of [C_4_mIm][MR] over the conventional Na^+^ salt of MR from pH 1 to 7, and **(bottom) **the increased selectivity of the [C_4_mIm][MR] (***right***) to HOTf over the conventional sodium salt in ethanol (***left***) [122] (Reproduced by permission of The Royal Society of Chemistry).

Zhang *et al.* further developed on the concept of azobenzene functionalized ILs in a separate publication. In this paper, ILs based on imidazolium and pyrrolidinium cations and methyl red (MR) were prepared for use as acido-responsive sensors in aqueous and non-aqueous solutions [122]. MR differs slightly to MO in that a carboxylate group replaces the sulfonate in position R_1_. The authors detail the advantages in preparing ILs with MR as shown in Figure 11.

The sodium salt of MR was shown to be sparingly soluble in water, meaning its pH responsive chemistry is void in this solvent. By exchanging sodium for [C_4_mIm]^+^, the resultant IL was completely miscible with water and the pH responsiveness of the chromophore allowed a selective response to be observed in pH’s ranging from 1–7 (Figure 11 (top)). 

Furthermore, an increased selectivity for the same IL over MR for trifluoromethanesulfonic acid (HOTf) was observed in solutions of ethanol (Figure 11 (bottom)). The authors postulate a differing response mechanism for the ILs response over MR and attribute it to the increased response to HOTf at low concentrations. 

This particular publication highlights the novelty and advantages of preparing ILs with stimuli responsive chromophores. The unique ionic nature of these liquids allows well-known chemistries to be performed in previously inhospitable solvent environments.

### 5.2. Electrochromic ILs

**Figure 12:**
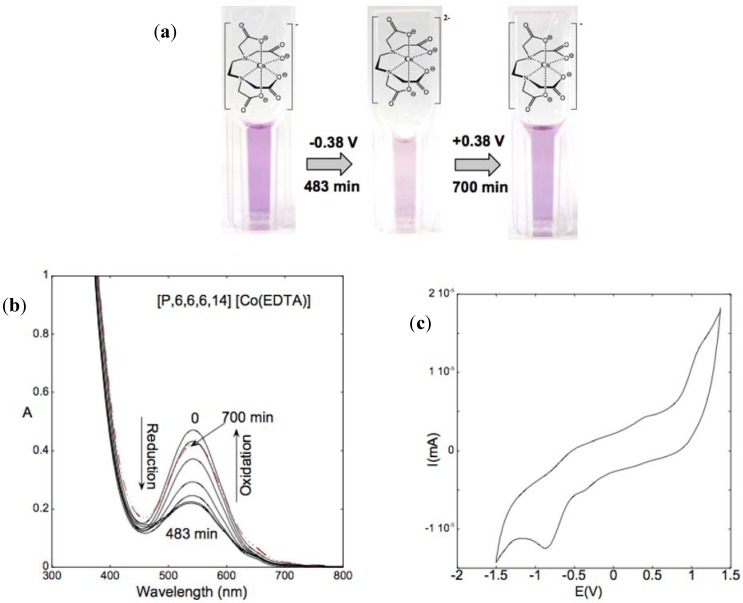
**(a) **The reversible optical redox chemistry of [P_6,6,6,14_][Co(EDTA)]. **(b) **Change in absorbance as a function of the applied voltage and (**c) **the CV obtained for of [P_6,6,6,14_][Co(EDTA)] [123] (Reproduced by permission of The Royal Society of Chemistry).

A recent communication by Pina *et al.* described the syntheses of intrinsically electrochromic ILs based on the transition metal complexes of ethylenediaminetetraacetic acid (EDTA) [123]. Again the ILs were prepared via ion-exchange of its Na^+^ salt with the relevant halide salt of cations based on phosphonium, imidazolium and ammonium derivatives. The spectroelectrochemical response obtained for the phosphonium IL is shown below in Figure 12. 

In this case the electrochromic functionality of the IL is a result of the coordinated central atom switching between two distinct redox states (Co^2+/3+^). A secondary electrolyte was not needed as the intrinsic conductivity of the IL facilitated the current between two electrodes. 

## 6. Conclusions

Our overview has attempted to present the current state of development in the field of employing stimuli responsive ionogels as active components in chemical sensors, as reported in the open literature. Arguably the most advantageous feature of using ionogels within sensing templates is the wide degree of ion choice, and related breadth of tunability of the properties of the IL and therefore the behavior of ionogels that are based on them (be it either the cation or anion of the IL, or the chemical structure of the repeating unit). We have highlighted the applications of ionogel chemistry in diverse research areas such as photo-controlled valves in microfluidic devices, as active components in optical/electrochemical sensors and in the development of robust bio-chemical sensing platforms. We have also highlighted a new emerging area in the development of stimuli responsive ILs, where the responsive chemistries of existing materials are integrated into an IL in the liquid phase. 

The use of ionogels in sensing platforms clearly has several advantages over current technologies. There is great potential in the development of ionogel science, with a diverse area of application ready to be explored. 
